# Development of a High-Temperature and High-Salinity Resistant Branched Polymer for Controlling Fluid Loss of Water-Based Drilling Fluids

**DOI:** 10.3390/gels12090782

**Published:** 2026-09-01

**Authors:** Lili Yan, Ren Wang, Kaihe Lv, Taifeng Zhang, Xiaoxiao Ni, Duanni Li, Yawen Li, Haijun Yang

**Affiliations:** 1 CNPC Engineering Technology R&D Company Limited, National Engineering Research Center of Oil & Gas Drilling and Completion Technology, Beijing 102206, China; 2 School of Petroleum Engineering, China University of Petroleum (East China), Qingdao 266500, China

**Keywords:** fluid-loss reducer, branched polymer, high-temperature, high-salinity

## Abstract

Conventional fluid-loss reducers lack thermal and salt resistance and exhibit a pronounced viscosity-enhancing effect in bentonite-based drilling fluids. These issues will cause severe problems during drilling operations. Herein, a branched fluid-loss reducer, BPJ, was synthesized using BPEI1_800_ as the branching core and DMAA, AMPS, DMDAAC, and AN as monomers. BPJ showed good thermal stability, with less thermal degradation at 235 °C. BPJ was thermally stable up to 220 °C in drilling fluids, and it could tolerate saturated NaCl, 10% KCl, and 5% CaCl_2_. Compared with commercial fluid-loss additives, BPJ had better fluid-loss reduction and lower viscosity enhancement. Under 220 °C and 36 wt% NaCl conditions, BPJ adsorbed strongly onto bentonite surfaces. Sulphonate groups increased electrostatic repulsion and maintained good colloidal stability of the bentonite. It further inhibited aggregation and detrimental dispersion of bentonite particles. BPJ was synergistic with bentonite, helping to form an impermeable filter cake and resulting in efficient filtration loss control. The study presents a novel approach to developing fluid-loss reducers for high-temperature, high-pressure drilling fluids in deep and ultra-deep formations.

## 1. Introduction

As the exploration of petroleum progressively expands toward deep and ultra-deep formations, drilling wells deeper than 6000 m has become increasingly important for hydrocarbon development [1,2,3]. Drilling fluids perform several essential functions throughout the drilling process, such as controlling formation pressure, stabilizing the wellbore, transporting cuttings to the surface, and lubricating the drill bit. Consequently, maintaining stable properties of drilling fluids is essential for the success of drilling engineering [4].

Increasing well depth exposes drilling fluids to increasingly harsh downhole environments, where formation temperatures commonly exceed 200 °C and may reach 220 °C. Under these conditions, conventional fluid-loss reducers can lose effectiveness because of limited thermal and salt resistance, resulting in unstable rheological behavior and inadequate filtration control. Such deterioration increases the risks of wellbore collapse, stuck-pipe incidents, and blowouts, posing a major challenge to the safe and efficient exploitation of hydrocarbon resources [5,6].

Fluid-loss reducers are mainly classified into natural modified polymers and synthetic polymers. Gorlov developed a fluid-loss reducer, LTTA, using brown coal as the raw material. The thermal stability of LTTA could reach 165 °C [7]. Amanullah et al. [8] prepared five different starch-based fluid-loss reducers. When the starch was 0.57% in drilling fluids, the FL_API_ was 22 mL after aging at 175 °C. Wang [9] introduced water-soluble sodium silicate into sodium carboxymethyl starch to synthesize an organosilicon compound fluid-loss reducer. After aging at 150 °C for 16 h, the FL_API_ was 15.2 mL when 0.4% polymer was added. Natural and naturally modified fluid-loss reducers contain weak bonds such as ether bonds and glycosidic bonds. They will fail in ultra-high-temperature (220 °C) formations. Synthetic polymers are highly versatile with adjustable types and quantities of functional groups and molecular weights. Fluid-loss reducers with good temperature resistance can be prepared by copolymerizing various vinyl monomers. Perricone prepared two copolymers, COP-1 and COP-2, using AM and AMPS as monomers. COP-1 and COP-2 could be stable under 177 °C. Nagre [10] developed two copolymers, CP4 and CP5, through the copolymerization of AM, AMPS, N-vinylcaprolactam, and vinyl acetate. Both CP4 and CP5 remained effective at temperatures up to 150 °C. Ma et al. [11] reported a betaine copolymer, PAASD, that tolerated 200 °C and 35 wt% salt. After adding 2 wt% PAASD to the base slurry, the AV was 63.5 mPa·s, and the PV was 42.0 mPa·s. Wang et al. [12] prepared a fluid-loss reducer, PFS-1, with multi-site-adsorbing groups. PFS-1 could retain its performance at 220 °C and in 35% NaCl brine. However, incorporating 2.0 wt% PFS-1 increased the AV of the base slurry to 85.0 mPa·s. Li et al. [13] synthesized a hyperbranched fluid-loss reducer, HBPSiO-DADV, by introducing a hyperbranched polysiloxane structure. HBPSiO-DADV remained stable at 200 °C and was compatible with saturated NaCl. At a dosage of 3.0 wt%, the AV and PV of the HBPSiO-DADV-based slurry were 45.0 mPa·s and 33.0 mPa·s, indicating that the hyperbranched architecture produced a comparatively moderate viscosity enhancement in freshwater-based drilling fluids.

Although many fluid-loss reducers are available, most additives lack resistance to ultra-high temperature (220 °C) and high salinity (36% NaCl). Linear polymers resist temperatures up to 220 °C, but they generally cause a significant increase in viscosity in freshwater-based slurries, which may lead to excessive viscosity. This makes drilling fluids difficult to pump. To overcome the limitations of conventional additives, a branched polymeric fluid-loss reducer (BPJ) was synthesized from N,N-dimethylaminopropyl acrylamide (DMAA), dimethyldiallylammonium chloride (DMDAAC), 2-acrylamido-2-methylpropane sulfonic acid (AMPS), and acrylonitrile (AN) in the presence of BPEI_1800_. BPJ retained its functionality at temperatures up to 220 °C and under saturated-salt conditions. Compared with its linear counterpart, BPJ caused less viscosity enhancement in freshwater-based mud while providing effective filtration control, demonstrating its potential for use in water-based drilling fluids in deep and ultra-deep wells.

## 2. Results and Discussion

### 2.1. Characterization of BPJ

**Figure 1:**
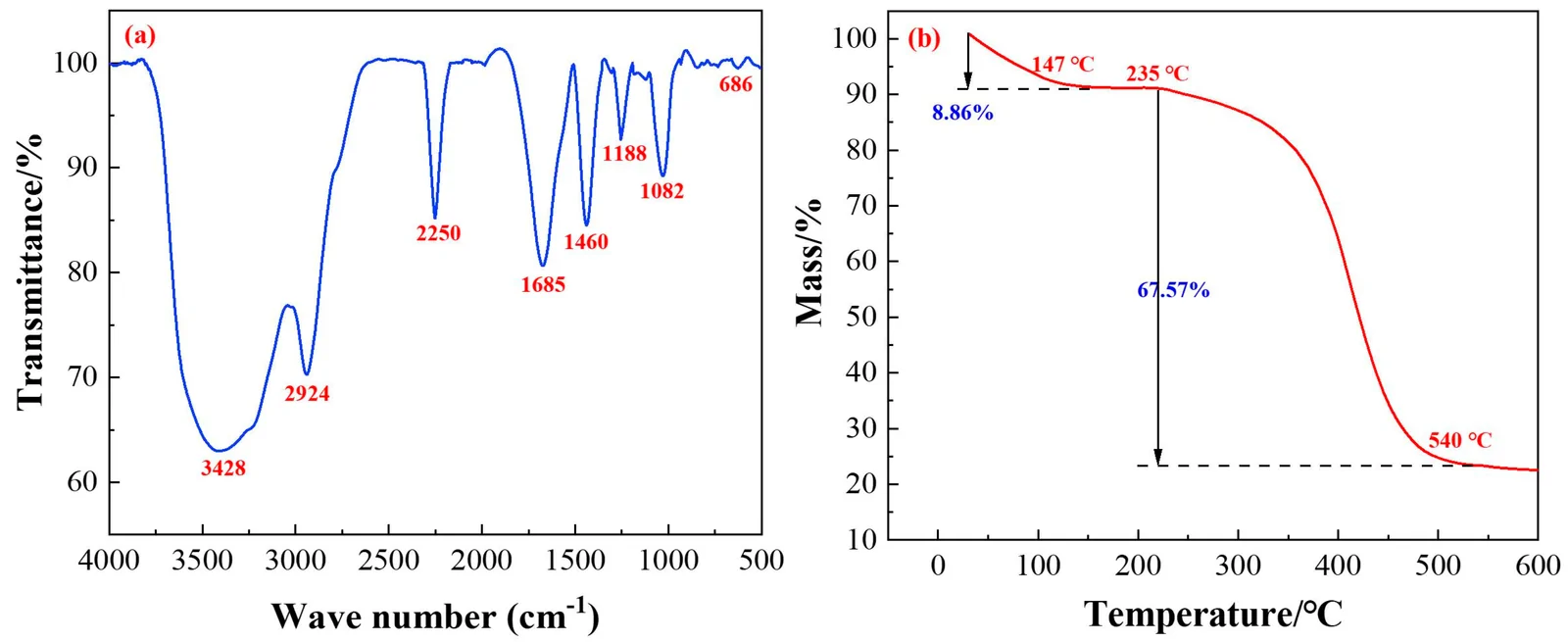
Characterization of BPJ: (**a**) infrared spectrum diagram of BPJ; (**b**) thermogravimetric analysis of BPJ.

As shown in Figure 1a, the peak centered at 3428 cm^−1^ was assigned to the N–H stretching of amide moieties, whereas the signal at 2924 cm^−1^ arose from asymmetric methylene stretching [14]. The band near 2250 cm^−1^ was characteristic of C≡N stretching, demonstrating the introduction of acrylonitrile units into the polymer. In addition, the absorption at 1685 cm^−1^ was associated with the C=O stretching of amide groups, while that at 1460 cm^−1^ was attributed to the quaternary ammonium functionality. The bands located at 1188 and 1082 cm^−1^ originated from the S=O and S–O vibrations of the sulfonic acid moieties, respectively [15], and the signal at 626 cm^−1^ was assigned to C–S stretching [16]. Collectively, these spectral features verified the presence of functional units derived from all four monomers, supporting the successful preparation of BPJ.

The thermogravimetric profile of BPJ in Figure 1b can be separated into three regions over 30–600 °C. Between 30 and 147 °C, BPJ underwent an 8.86% mass reduction, mainly because of the removal of free water and bound water associated with the amide and sulfonic acid functionalities. Little additional mass change occurred between 147 and 235 °C, suggesting that the polymer framework remained relatively stable in this interval. The principal decomposition region extended from 235 to 540 °C, accompanied by a 67.57% mass loss resulting from the degradation of amide, sulfonic acid, quaternary ammonium, and nitrile-containing units [17]. Above 540 °C, only a limited mass variation was observed, corresponding to the gradual cleavage and carbonization of the remaining C–C backbone [18]. The incorporation of thermally stable sulfonic acid and nitrile functionalities contributed to BPJ’s thermal resistance. Its limited decomposition below 235 °C supports its potential to be a fluid-loss reducer in ultra-deep-well drilling fluids.

**Figure 2:**
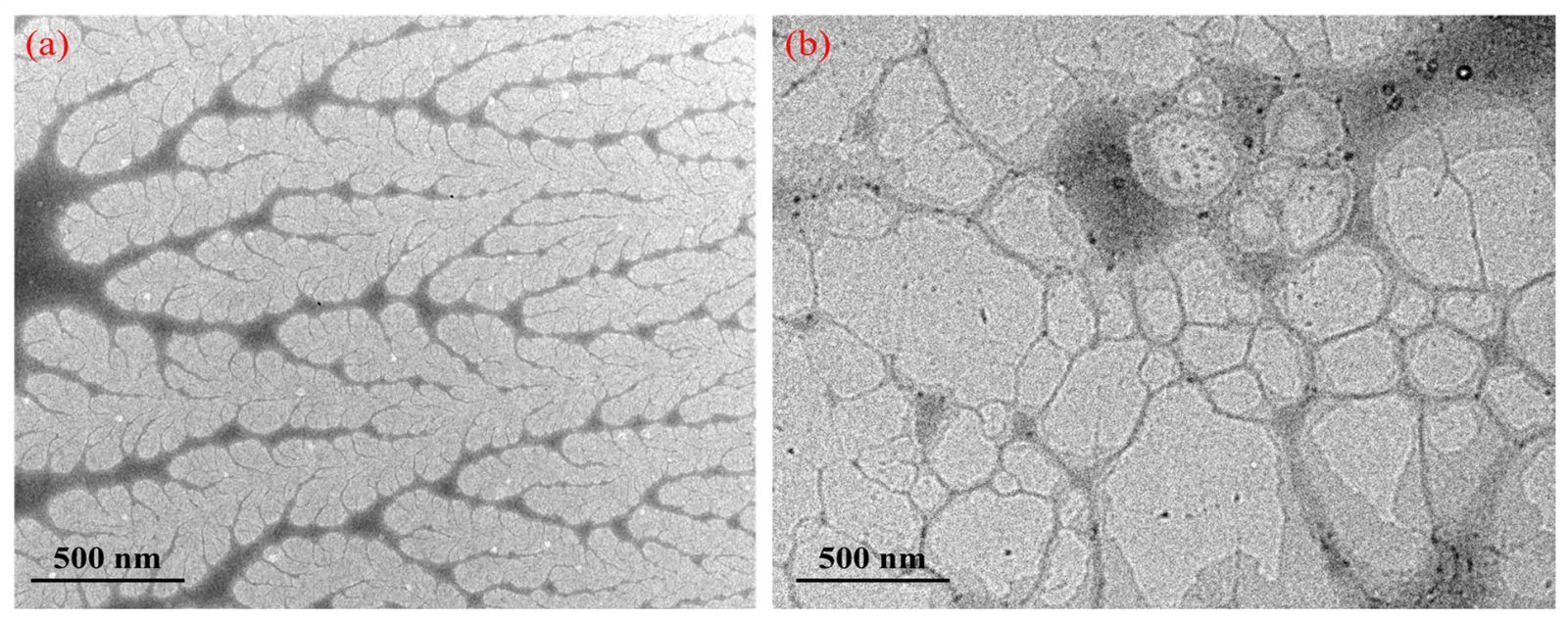
Microscopic morphology of polymers: (**a**) LPJ; (**b**) BPJ.

As shown in Figure 2a, LPJ displays a linear molecular structure. The linear molecular chains of LPJ possess greater conformational freedom and can extend over longer distances, thereby facilitating intermolecular association and chain entanglement. In contrast, BPJ exhibited a relatively compact and discrete microstructure, characterized by numerous irregular polygonal or quasi-spherical domains. These domains are distributed relatively uniformly and show limited long-range interconnection. This morphology is consistent with BPJ’s highly branched molecular architecture, in which abundant branch points restrict excessive chain extension and intermolecular entanglement. Consequently, BPJ exhibits a smaller effective hydrodynamic volume and weaker chain entanglement, thereby resulting in a lower viscosity-increasing effect than LPJ at the same concentration.

The molecular-weight characteristics of BPJ and LPJ are summarized in Table 1. BPJ exhibited a weight-average molecular weight (M_w_) of 463,776 g·mol^−1^ and a number-average molecular weight (M_n_) of 257,331 g·mol^−1^, with a polydispersity index (PDI) of 1.802. In comparison, LPJ showed slightly higher M_w_ and M_n_ values of 506,877 and 267,582 g·mol^−1^, respectively, with a PDI of 1.894. The comparable molecular weights of BPJ and LPJ indicate that the pronounced difference in their viscosity-increasing behavior is more closely associated with their distinct molecular architectures rather than molecular-weight differences.

### 2.2. Performance Evaluation of BPJ

#### 2.2.1. Effect of BPJ Concentration on the Properties of FWBDFs

**Figure 3:**
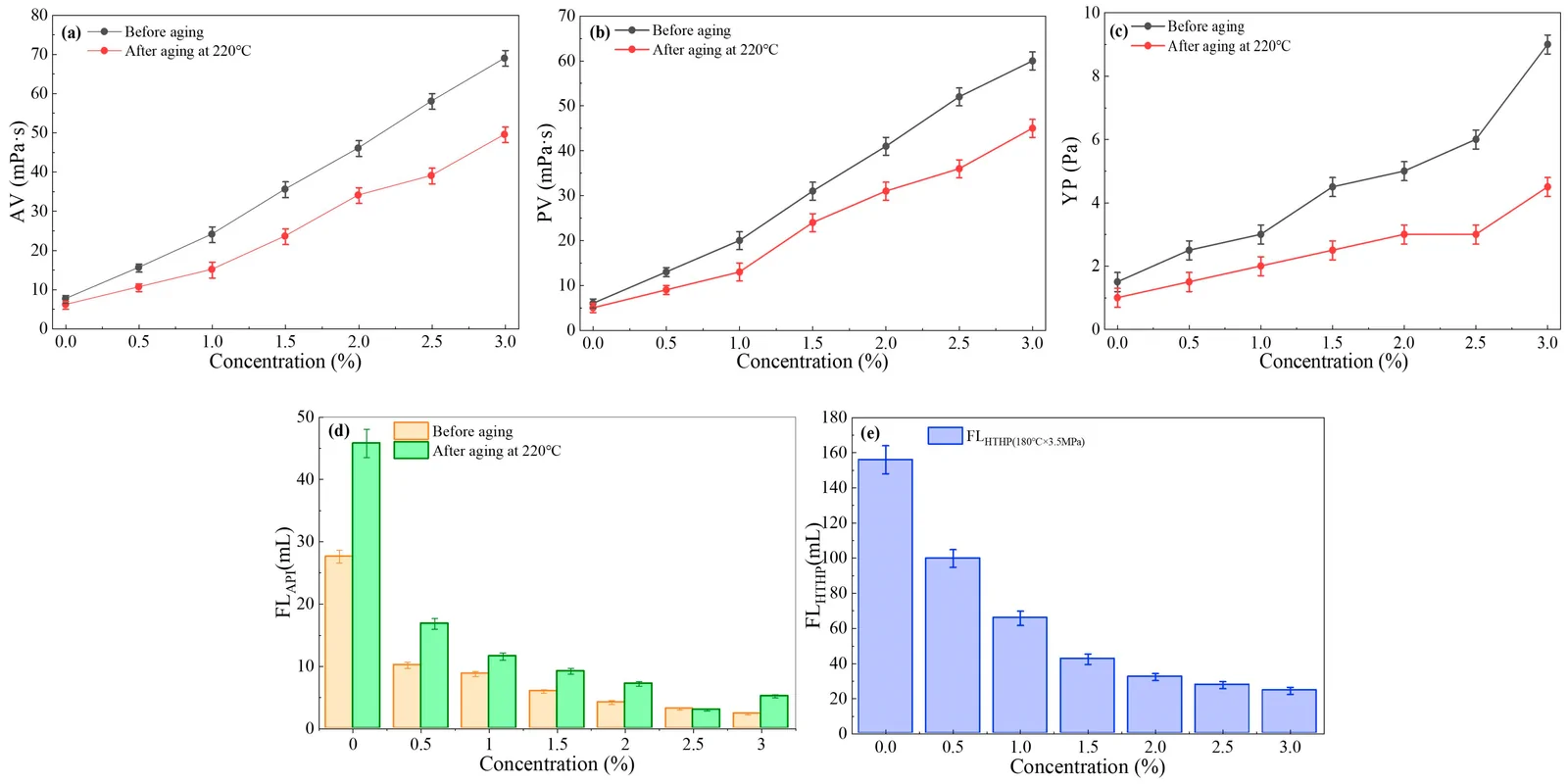
Effect of BPJ on the performance of FWBDFs: (**a**) AV; (**b**) PV; (**c**) YP; (**d**) FL_API_; (**e**) FL_HTHP_.

As shown in Figure 3, increasing the BPJ concentration progressively raised the AV, PV, and YP, while both FL_API_ and FL_HTHP (180 °C, 3.5 MPa)_ declined. Nevertheless, the viscosity enhancement remained controlled within the investigated dosage range. At 2.0 wt% BPJ, the AV and YP were 46.0 mPa·s and 5.0 Pa, respectively, and increased to 58.0 mPa·s and 6.0 Pa at 2.5 wt%. The FL_HTHP (180 °C, 3.5 MPa)_ was 32.4 and 27.8 mL after aging at 220 °C. BPJ could provide sufficient viscosity and structural strength to improve cuttings suspension and transport without causing pronounced thickening. Meanwhile, interactions between BPJ and clay particles facilitate particle packing and the development of a dense filter cake, thereby improving filtration control. When the BPJ dosage was 3.0 wt%, it resulted in excessive fluid viscosity but produced only marginal additional reductions in FL_API_ and FL_HTHP (180 °C, 3.5 MPa)_. Considering the balance between rheological performance and fluid-loss control, the optimal BPJ concentration for FWBDFs was 2.5 wt%.

#### 2.2.2. Temperature Resistance

**Figure 4:**
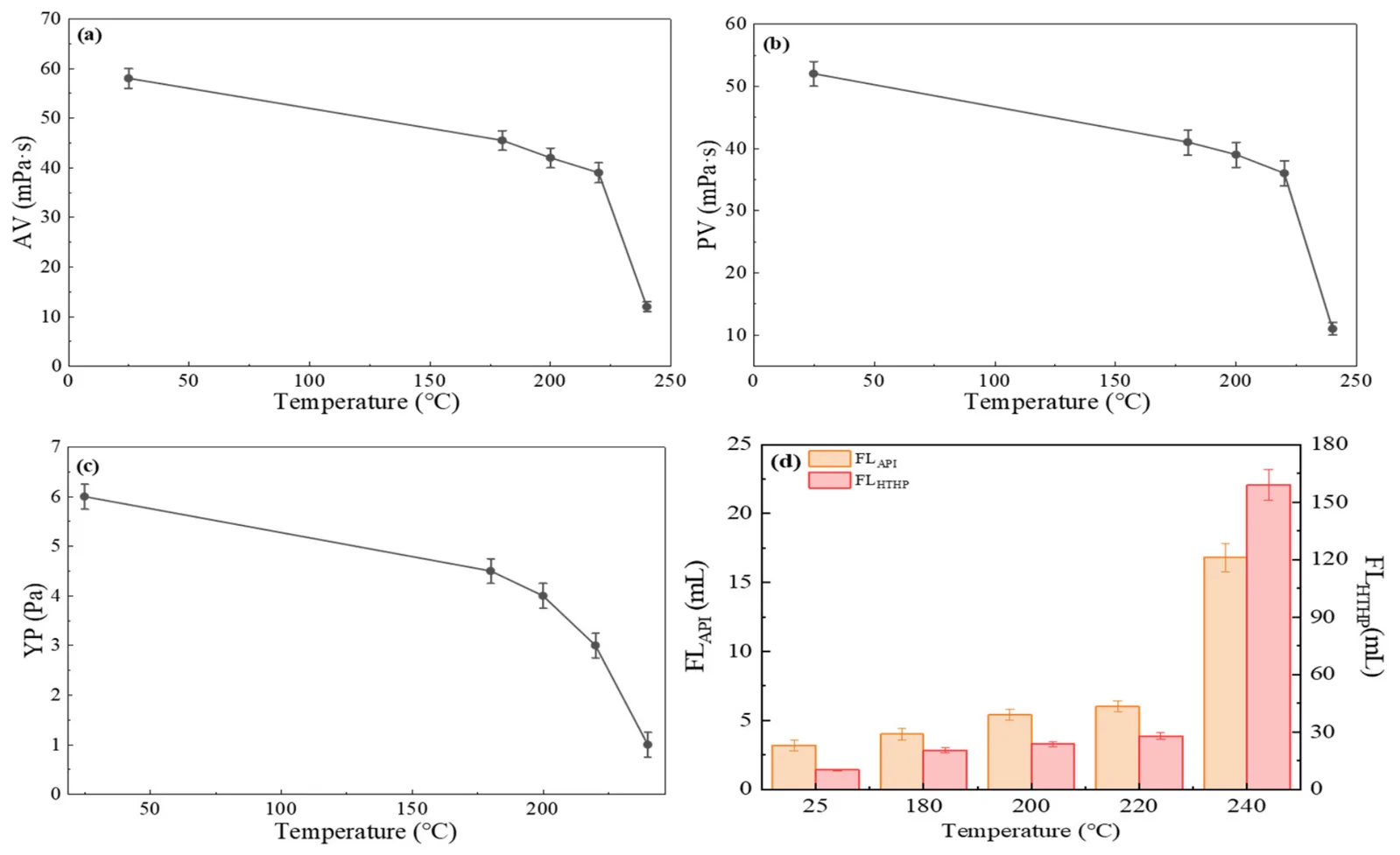
Temperature resistance of BPJ in FWBDFs: (**a**) AV; (**b**) PV; (**c**) YP; (**d**) FL_API_ and FL_HTHP_. (**e**) FL_HTHP_.

The results are shown in Figure 4. The BPJ-FWBDFs still have appropriate rheological properties and fluid loss after aging at 220 °C, demonstrating the good thermal stability of BPJ. In contrast, there was a pronounced deterioration in the performance of BPJ-FWBDFs as the aging temperature increased to 240 °C, as evidenced by the sharp reductions in AV, PV, and YP. This suggested that significant thermal degradation of BPJ occurred at 240 °C, severely impairing its ability to maintain viscosity and control fluid loss. Consequently, the maximum effective temperature resistance of BPJ was determined to be 220 °C.

#### 2.2.3. Salt Resistance

**Figure 5:**
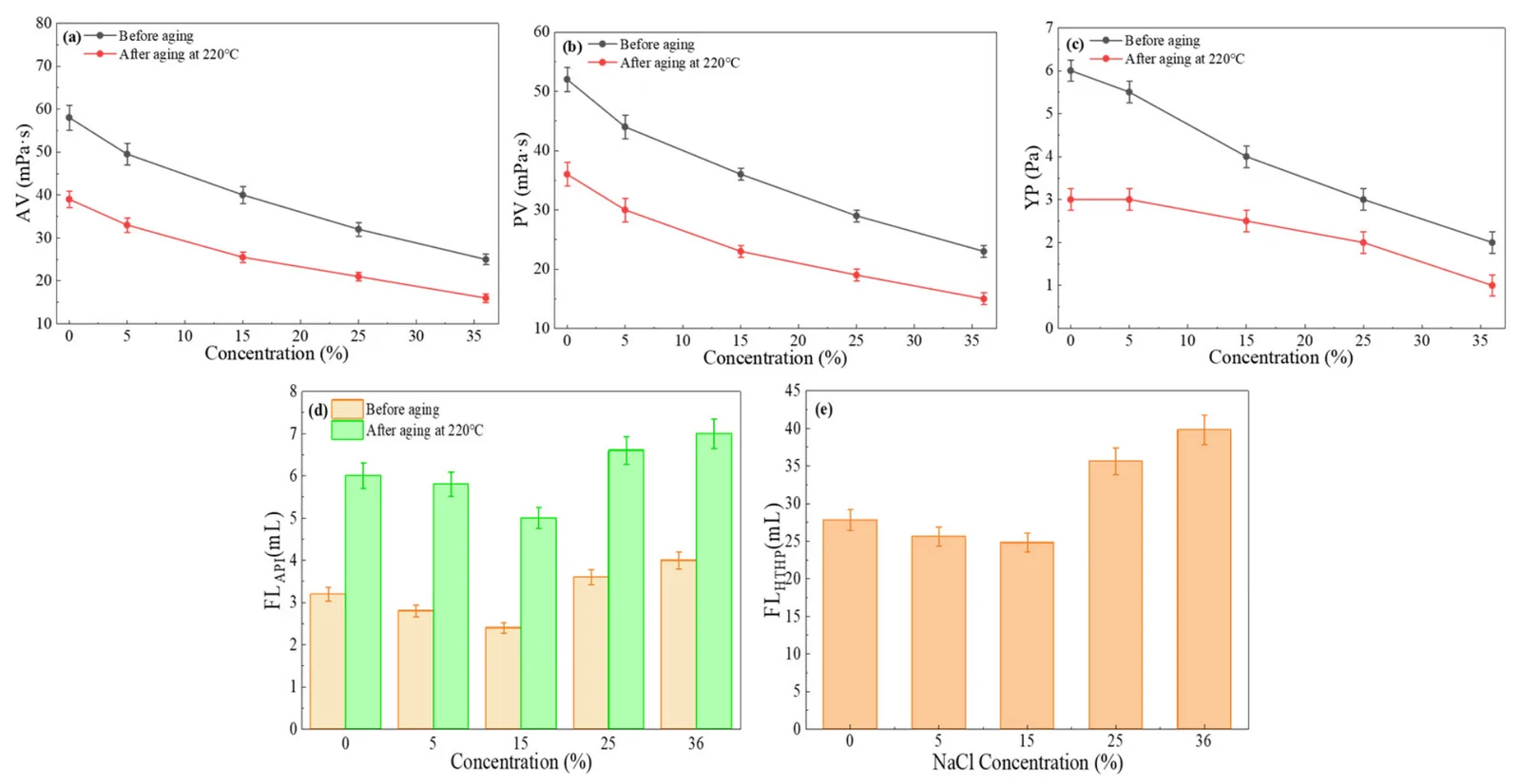
NaCl resistance of BPJ under 220 °C: (**a**) AV; (**b**) PV; (**c**) YP; (**d**) FL_API_; (**e**) FL_HTHP_.

The results are shown in Figure 5. As the NaCl concentration increased, the AV, PV, and YP declined continuously. This rheological deterioration can be associated with the accumulation of Na^+^ at the bentonite–water interface, which reduces the number of surface sites available for BPJ adsorption. Meanwhile, ionic screening weakens the electrostatic association between BPJ and bentonite particles, leading to partial disruption of the internal network structure [19]. In contrast, FL_API_ and FL_HTHP (180 °C, 3.5 MPa)_ initially increased and subsequently decreased with increasing salinity. This nonmonotonic behavior arose from the competing effects of NaCl on BPJ. Salt ions can screen the internal ionic associations between sulfonate and quaternary ammonium groups, making these charged functionalities more accessible for interaction with clay surfaces and partially restoring BPJ adsorption. This effect helps preserve clay hydration and dispersion stability. At very high ionic strength, however, pronounced charge screening and electrical-double-layer compression reduce polymer-chain expansion and partially impair BPJ performance. Even in a saturated NaCl solution containing 36% salt, the FL_HTHP (180 °C, 3.5 MPa)_ remained at 39.8 mL after aging at 220 °C, demonstrating that BPJ retained effective fluid loss control under high-temperature and high-salinity conditions.

**Figure 6:**
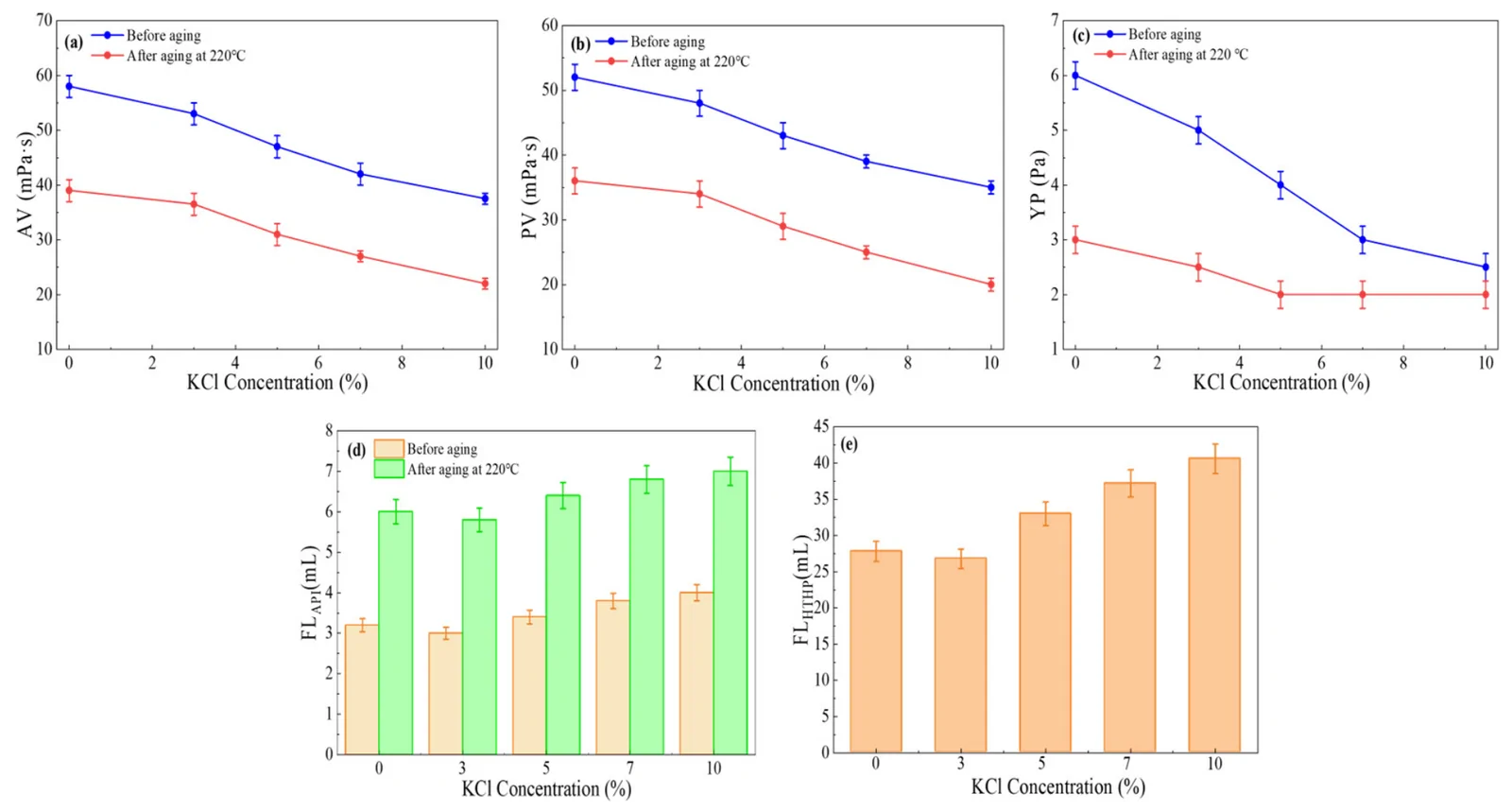
KCl resistance of BPJ under 220 °C: (**a**) AV; (**b**) PV; (**c**) YP; (**d**) FL_API_; (**e**) FL_HTHP_.

The results are shown in Figure 6. Increasing the KCl concentration from 0% to 10% progressively reduced the AV, PV, and YP. Similar to Na^+^, K^+^ weakens the associations between BPJ and bentonite particles, thereby disrupting the network responsible for the fluid’s rheological properties [20]. By contrast, FL_API_ and FL_HTHP (180 °C, 3.5 MPa)_ followed a nonmonotonic trend, decreasing initially and subsequently increasing with KCl concentration. At 10% KCl, the FL_HTHP (180 °C, 3.5 MPa)_ measured after aging at 220 °C was 40.6 mL. Thus, despite the salt-induced deterioration in rheological properties, BPJ retained effective fluid-loss control after exposure to 220 °C and 10% KCl.

**Figure 7:**
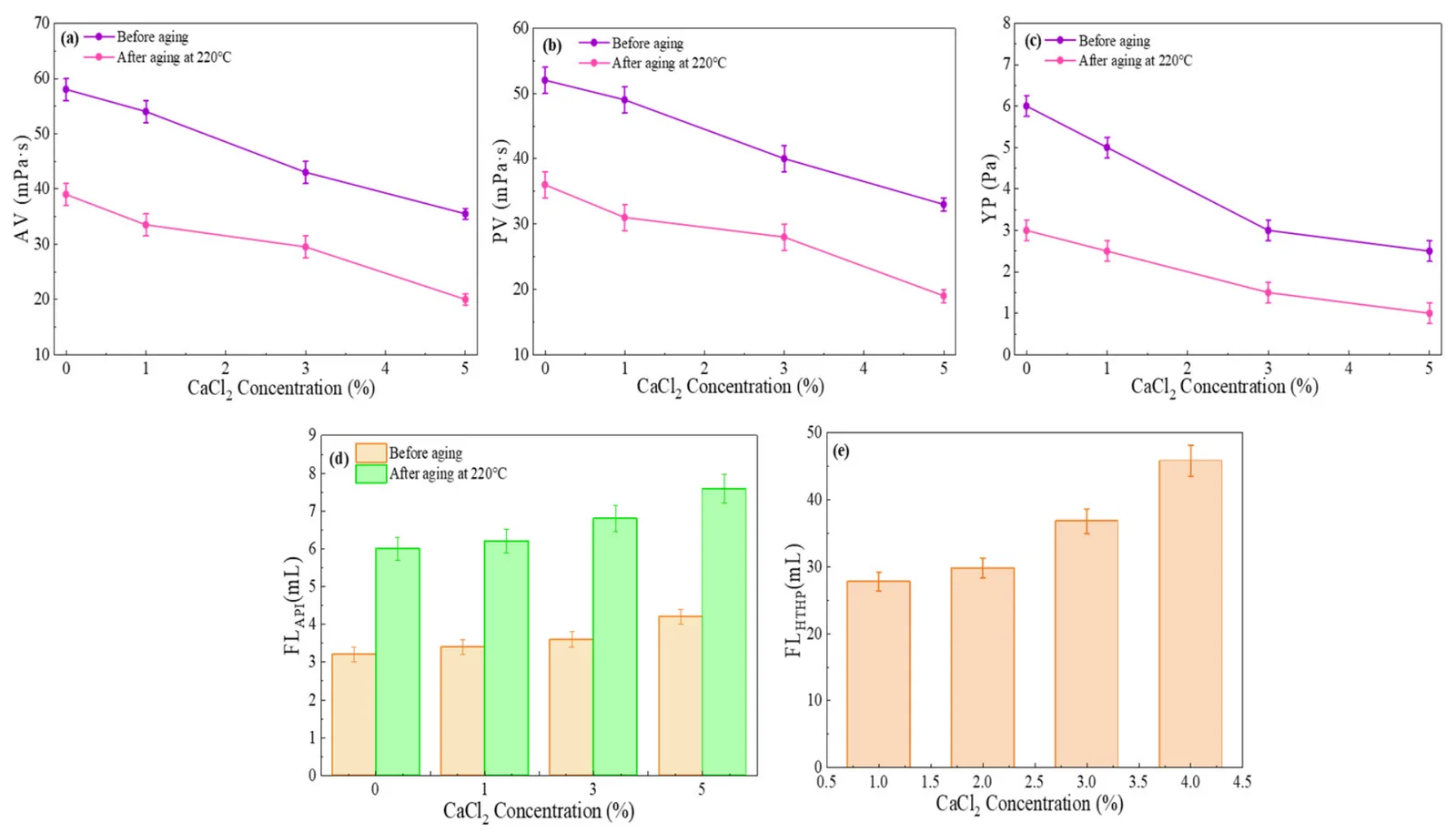
CaCl_2_ resistance of BPJ under 220 °C: (**a**) AV; (**b**) PV; (**c**) YP; (**d**) FL_API_; (**e**) FL_HTHP_.

The results are shown in Figure 7. The divalent cation Ca^2+^ exhibits a stronger ability to compress the diffuse electrical double layers of bentonite particles and BPJ chains than monovalent cations, resulting in deterioration of clay colloidal stability [20]. Despite this adverse effect, 2.5 wt% BPJ maintained the FL_HTHP (180 °C, 3.5 MPa)_ of only 45.8 mL after aging at 220 °C in the presence of 5 wt% CaCl_2_. This finding indicates that BPJ exhibited good calcium resistance.

#### 2.2.4. Comparison to Linear Polymer LPJ

The performance of the branched polymer BPJ and the linear polymer LPJ was compared in both FWBDFs and SWBDFs. The results are shown in Table 2 and Table 3. When both BPJ and LPJ were added at 2.5 wt% to the FWBDFs, the AV and PV of the LPJ-FWBDFs significantly increased to 106.0 mPa·s and 96.0 mPa·s. A pronounced viscosity-enhancing effect of LPJ on FWBDFs was indicated. In contrast, the AV and PV of BPJ-FWBDFs were 58.0 mPa·s and 52.0 mPa·s, suggesting that the unique branched structure of BPJ endows it with a smaller hydrodynamic volume and a reduced viscosity-enhancing effect [21]. The AV and PV of the BPJ-SWBDFs remained lower than those of the LPJ-SWBDFs. Regarding fluid-loss performance, BPJ exhibited slightly superior reductions in both FL_API_ and FL_HTHP (180 °C, 3.5 MPa)_ compared with LPJ. The results indicate that BPJ provides less viscosity enhancement and more effective fluid-loss control than the linear polymer LPJ.

#### 2.2.5. Comparison to Other Fluid-Loss Reducers

For a more reasonable comparison, the commercial additives were evaluated under identical drilling fluid formulations, aging conditions, and testing procedures as BPJ.

The performance comparison of different fluid-loss reducers is shown in Table 4. At a concentration of 2.5 wt%, DS-2 and Driscal D significantly increased the viscosity of the SWBDFs. The PV of DS-2-SWBDFs and Driscal D-SWBDFs was 47.0 mPa·s and 84.0 mPa·s. By comparison, DS-1 and BPJ exhibited much lower viscosity-enhancing effects, with PV values of 32.0 mPa·s and 23.0 mPa·s. Interestingly, SMP-1 showed a tendency toward post-aging thickening, as evidenced by an increase in viscosity after thermal aging. From a rheological perspective, BPJ and DS-1 were better suited to maintaining desirable rheological properties in high-density drilling fluids. With respect to fluid-loss control, DS-2, Driscal D, and SMP-1 displayed relatively weak fluid-loss reduction performance, as reflected by their higher FL_HTHP (180 °C, 3.5 MPa)_ values. In contrast, BPJ exhibited the lowest FL_HTHP (180 °C, 3.5 MPa)_ among all tested additives. Therefore, BPJ demonstrated the best fluid-loss reduction capability at 220 °C and under saturated salinity conditions [22].

### 2.3. Adsorption Capacity Analysis

**Figure 8:**
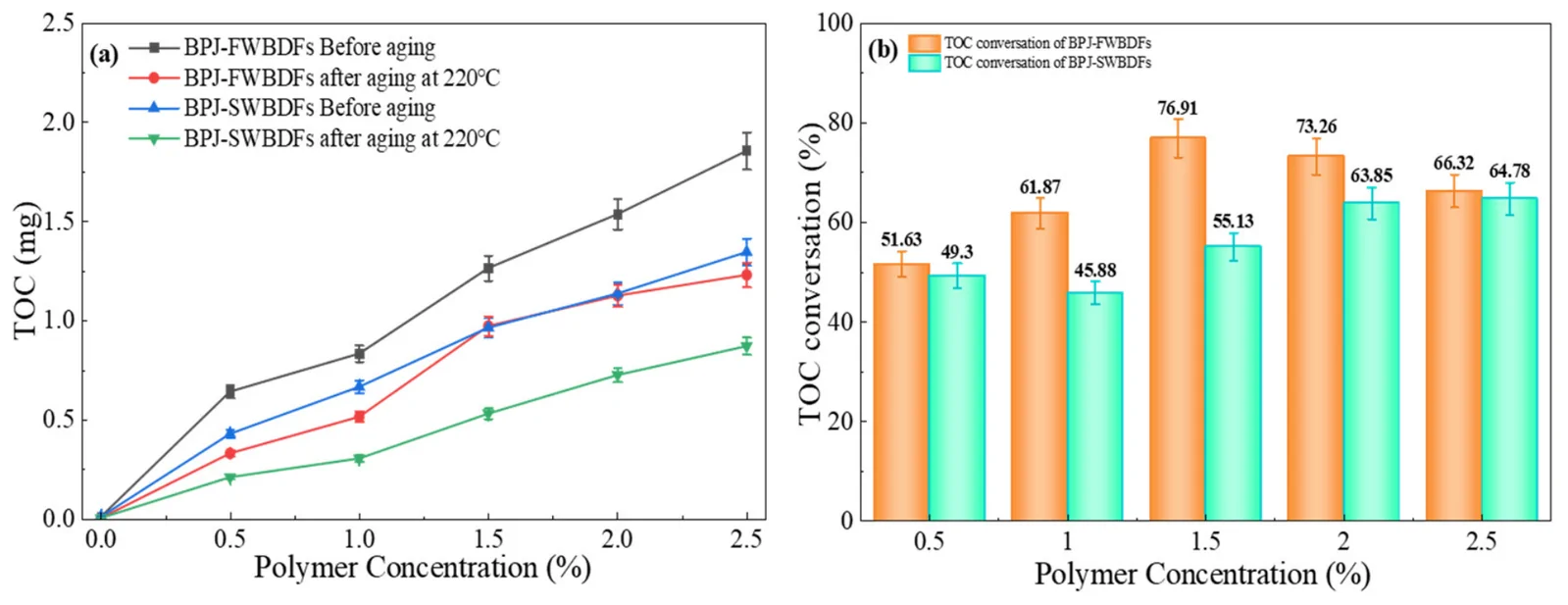
Adsorption capacity of BPJ before and after aging at 220 °C: (**a**) TOC of BPJ-FWBDFs and BPJ-SWBDFs; (**b**) TOC conversion of BPJ-FWBDFs and BPJ-SWBDFs.

Na^+^ easily caused polymer curling in high-salinity environments. Elevated temperatures induced degradation of the polymer backbones. Both would decrease the adsorption capacity of polymers [23,24]. Therefore, fluid-loss reducers must exhibit good adsorption performance. As illustrated in Figure 8a,b, regardless of whether in FWBDFs or SWBDFs, the adsorption capacity of BPJ gradually increased with increasing BPJ concentration, indicating that BPJ could effectively adsorb on the clay surface. When the BPJ concentration was 2.5%, the TOC of BPJ reached 1.856 and 1.231 mg in FWBDFs before and after aging at 220 °C, with a TOC conversion of 66.32%. In a saturated NaCl environment, the TOC associated with adsorbed BPJ decreased from 1.346 mg before aging to 0.872 mg after exposure to 220 °C, corresponding to an adsorption-capacity retention of 64.78%. The remaining interfacial affinity was mainly attributed to the quaternary ammonium and amide functionalities of BPJ, which provide effective adsorption sites for interaction with clay. Therefore, BPJ maintained substantial adsorption on clay surfaces under the combined effects of 220 °C and saturated salinity, thereby preserving colloidal stability and reducing fluid loss [25].

### 2.4. Zeta Potential Analysis

**Figure 9:**
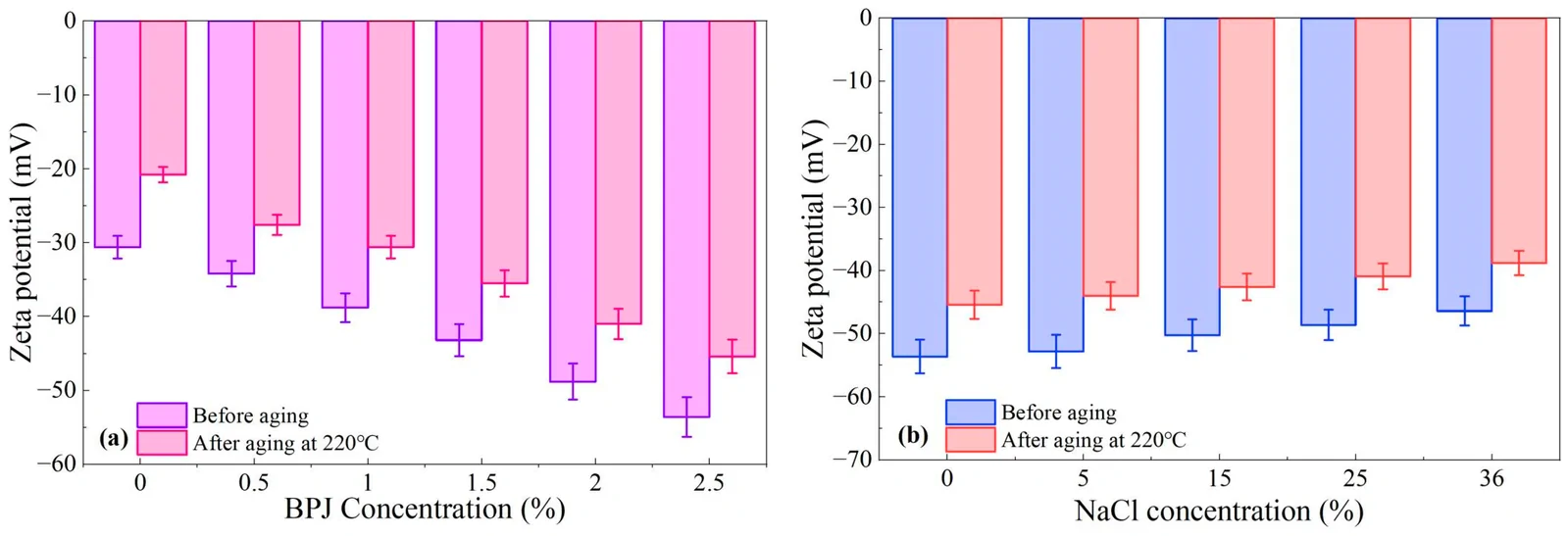
Zeta potential of BPJ-FWBDFs and BPJ-SWBDFs before and after aging at 220 °C: (**a**) BPJ-FWBDFs; (**b**) BPJ-SWBDFs.

In water-based drilling fluids, hydrated bentonite particles form the principal dispersed phase. The release of exchangeable cations during hydration produces an electrical double layer around the negatively charged clay surfaces. Zeta potential is therefore commonly used to evaluate the dispersion stability of clay suspensions. A larger negative potential generally corresponds to stronger electrostatic repulsion among particles, which suppresses aggregation and sedimentation. By contrast, an increase in zeta potential promotes particle association and may undermine the drilling fluid’s properties [26]. As shown in Figure 9, increasing the BPJ dosage shifted the zeta potential toward more negative values. Without BPJ, the zeta potentials were −30.6 and −20.8 mV before and after aging at 220 °C. At a BPJ concentration of 2.5%, the corresponding values reached −53.6 and −45.4 mV. These changes support the adsorption of BPJ onto bentonite by increasing the surface charge density and strengthening electrostatic repulsion between clay particles. In saturated NaCl solution, abundant Na^+^ compresses the electrical double layer and screens the negative surface charge, resulting in zeta potentials of only −9.68 and −7.26 mV before and after aging, respectively. The addition of 2.5% BPJ restored these values to −46.4 and −38.8 mV. Thus, BPJ retained its ability to interact with clay surfaces even after exposure to 220 °C and saturated brine. The resulting improvement in dispersion stability facilitates the formation of an impermeable filter cake and consequently limits fluid invasion [27].

### 2.5. Size Distribution Analysis

**Figure 10:**
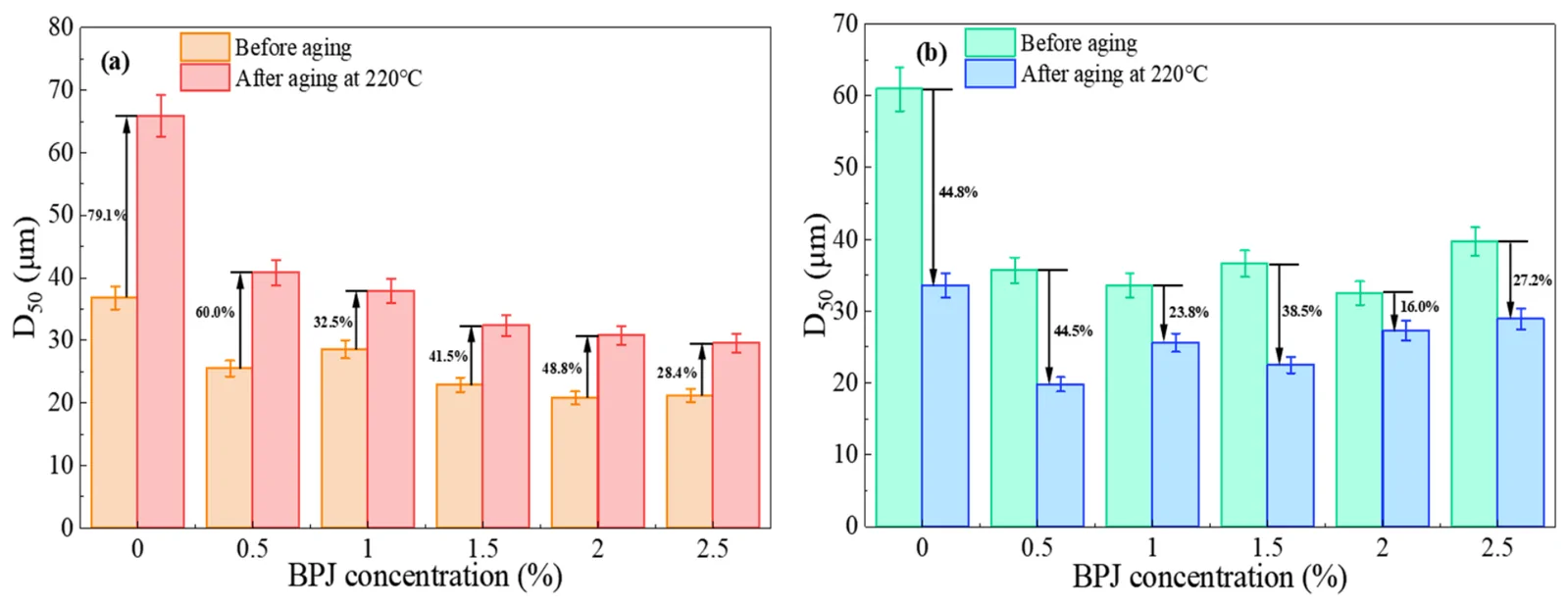
Size distribution analysis of BPJ-FWBDFs and BPJ-SWBDFs before and after aging at 220 °C: (**a**) BPJ-FWBDFs; (**b**) BPJ-SWBDFs.

The results are shown in Figure 10. After aging at 220 °C, the D_50_ of FWBDFs was 36.8 μm and 65.9 μm, respectively. This indicated that the clay underwent agglomeration under high-temperature conditions. High temperatures could break the structure of clay particles. The hydration film on the clay surface became thinner, and the zeta potential decreased [28]. As a result, the clay agglomerated due to reduced colloidal stability. When BPJ was added, the particle size decreased after aging at 220 °C. It is worth noting that the increment in particle size decreased progressively as the BPJ concentration increased. When BPJ adsorbed onto the clay surface, it formed a thicker hydration film, promoting the hydration and dispersion of clay particles. The thicker hydration film maintained colloidal stability and prevented agglomeration of clay particles [29]. Na^+^ will inhibit the hydration and dispersion of clay particles, leading to clay agglomeration. The D_av_ of SWBDFs with saturated NaCl increased to 60.9 μm before aging. After aging at 220 °C, the clay underwent severe dispersion, with D_50_ decreasing from 60.9 μm to 33.6 μm. This indicated that the SWBDFs contained fewer small particles and larger particles after aging at 220 °C. These results suggested that bentonite dispersed at elevated temperature and salinity, resulting in a particle size distribution that was inappropriate for forming a compact, low-permeability filter cake. Nevertheless, the D_50_ of BPJ-SWBDFs remained relatively stable. Under HTHP conditions, BPJ effectively preserved the colloidal stability of clay particles and inhibited clay dispersion and agglomeration, thereby facilitating the formation of a compact mud cake by maintaining an appropriate particle size distribution and reducing filtration loss [30].

### 2.6. Mud Cake Analysis

**Figure 11:**
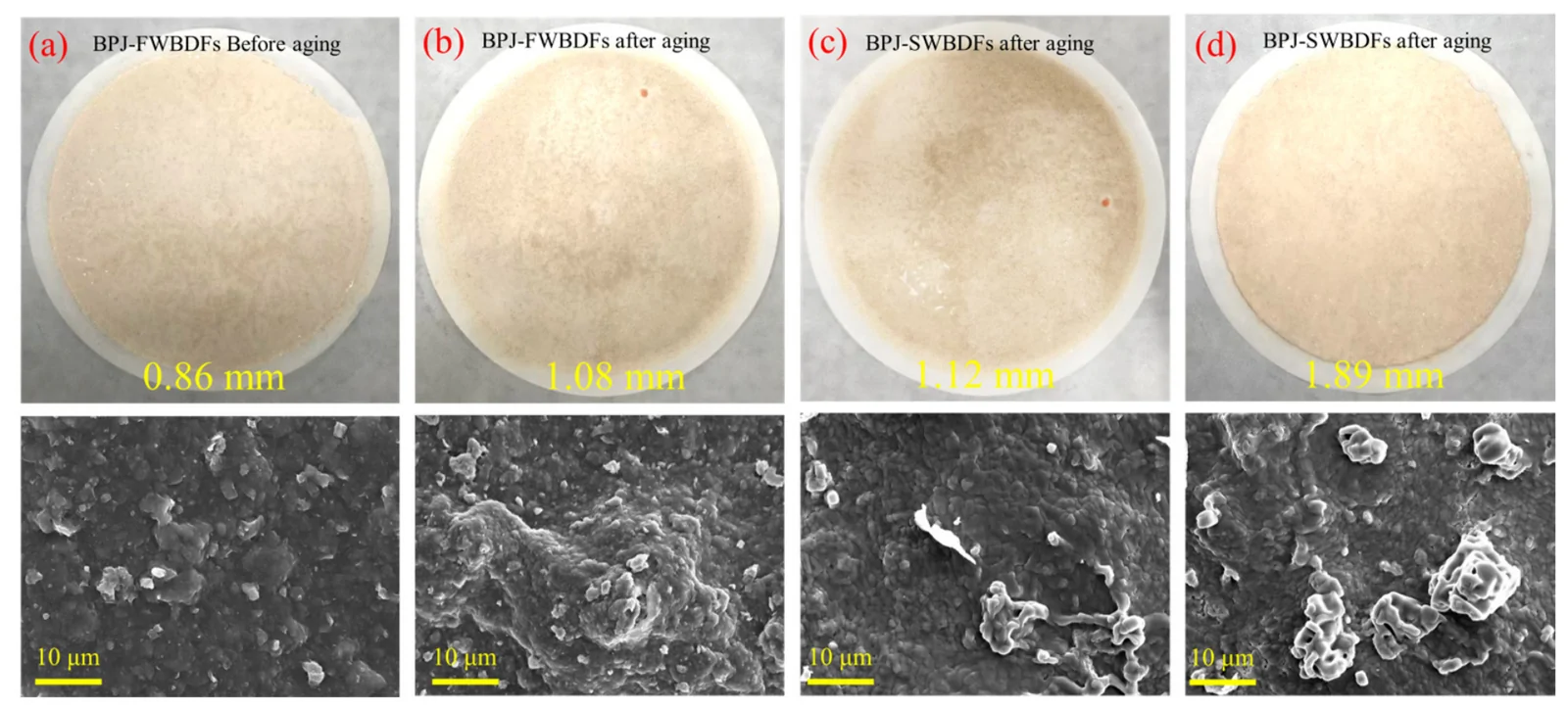
Morphology of mud cakes: (**a**) mud cake from BPJ-FWBDFs before aging; (**b**) mud cake from 3% BPJ-FWBDFs after aging at 220 °C; (**c**) mud cake from BPJ-SWBDFs before aging; (**d**) mud cake from BPJ-SWBDFs after aging at 220 °C.

As shown in Figure 11a,b, the BPJ-FWBDFs produced a compact cake with a thickness of 0.86 mm and no visible pores or cracks. Although some clay-particle aggregation occurred after aging at 220 °C, the resulting structure remained dense and contained no continuous pathways for filtrate invasion. Under saturated NaCl conditions, the cake shown in Figure 11c,d exhibits a uniform and compact surface with markedly fewer structural defects and a thickness of 1.12 mm. After aging at 220 °C, the BPJ-SWBDFs still formed a tightly packed filter cake without obvious pores or fractures, although its thickness increased to 1.89 mm. These morphological observations demonstrate that BPJ retains its functionality under simultaneous thermal and saline stresses. Its adsorption on clay surfaces helps stabilize the particles and maintain a size distribution favorable for close packing, thereby promoting the formation of a low-permeability filter cake and reducing fluid loss.

### 2.7. EDS Analysis

**Figure 12:**
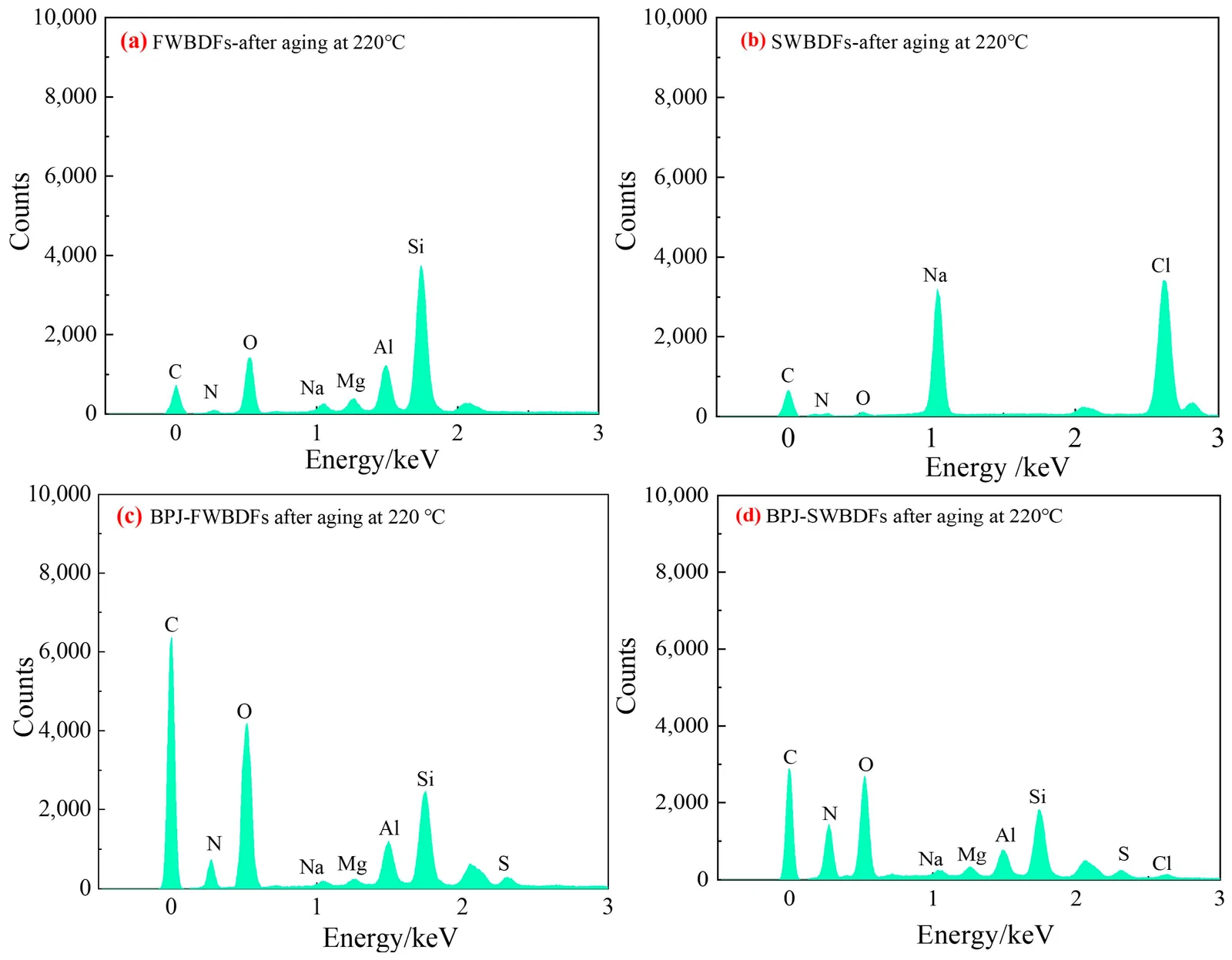
EDS elemental analysis of API filter cakes formed from (**a**) freshwater-based mud, (**b**) freshwater-based mud containing 2% BPJ, (**c**) saturated saltwater-based mud, and (**d**) saturated saltwater-based mud containing 2% BPJ.

From Figure 12a, it can be inferred that the mud cake obtained from FWBDFs was dominated by O, Si, and Al, whereas only weak signals of C, N, and Mg were detected. Incorporating 2% BPJ markedly increased the C, N, O, and S signals (Figure 12b), consistent with BPJ deposition on the clay surface. In saturated saltwater-based mud, aging at 220 °C produced substantial enrichment of Na and Cl, accompanied by very weak Si, Al, and Mg signals (Figure 12c). The accumulation of Na^+^ at the clay surface can screen the negative surface charge and weaken the colloidal stability of clay particles [31]. After 2% BPJ was introduced into the saturated-brine system, the C, N, O, and S contents increased again (Figure 12d), indicating that BPJ retained an appreciable affinity for the clay surface under HTHP conditions. This persistent adsorption contributes to clay-particle stabilization and improved filtration control.

## 3. Conclusions

A branched polymeric fluid-loss reducer, BPJ, was developed to overcome the limited temperature and salt resistance of conventional additives while minimizing excessive viscosification in bentonite-based drilling fluids. FTIR analysis verified the presence of the expected functional groups, and TGA indicated that BPJ resisted thermal decomposition under a nitrogen atmosphere. An optimal dosage of 2.5 wt% was determined. BPJ remained effective at 220 °C and exhibited tolerance to saturated NaCl, 10 wt% KCl, and 5 wt% CaCl_2_. Under conditions of 220 °C and 36 wt% NaCl, BPJ provided the lowest viscosity enhancement and the best filtration control among the commercial additives evaluated. Interfacial characterization indicated that BPJ maintained appreciable adsorption on bentonite surfaces, helping preserve surface hydration and electrostatic repulsion between clay particles. Consequently, BPJ limited both severe particle aggregation and excessive dispersion, maintained a particle size distribution favorable for close packing, and promoted the formation of a dense, low-permeability filter cake. These combined effects substantially improved fluid-loss control under high-temperature and high-salinity conditions. From a practical application perspective, BPJ has potential advantages due to its multifunctional structure, which enables simultaneous improvements in filtration control, salt tolerance, and rheological stability. The synthesis route relies on conventional polymerization techniques and commercially available monomers, making scale-up feasible. Nevertheless, this study was conducted primarily under laboratory conditions, and further field tests are required to evaluate the adaptability of BPJ in complex geological environments with various contaminants and dynamic drilling conditions. Comprehensive evaluation of production cost, industrial-scale synthesis, and environmental impact remains necessary before field application.

## 4. Materials and Methods

### 4.1. Materials

N,N-Dimethylaminopropylacrylamide (DMAPAA, 99%), 2-acrylamido-2-methyl propane sulfonic acid (AMPS, 98%), and dimethyl diallyl ammonium chloride (DMDAAC, 60 wt% aqueous solution) were purchased from the Shanghai Aladdin Bio-Chemical Technology Co., Ltd. (Shanghai, China) Acrylonitrile (AN, 99%), ammonium cerium nitrate (CAN, 99%), and 2,2′-Azobis(2-methylpropionamidine) dihydrochloride (V50, 97%) were provided by the Shanghai Macklin Biochemical Co., Ltd. (Shanghai, China). Branched polyethyleneimine (BPEI_1800_, 98%, weight-average molecular weight 1800) was bought from the Shanghai Yuanye Bio-Technology Co., Ltd. (Shanghai, China). Sodium chloride (NaCl, 99%), sodium hydroxide (NaOH, 99%), and sodium carbonate (Na_2_CO_3_, 99%) were provided by Sinopharm Reagent Co., Ltd. (Shanghai, China). Bentonite was purchased from Xinjiang Zhongfei Xiazi Jie Bentonite Co., Ltd. (Xinjiang, China). Fluid-loss reducers DS-1 and DS-2 were purchased from Shandong Deshun Yuan Technology Co., Ltd. (Dongying, China).

### 4.2. Synthesis of Polymers

Free-radical copolymerization was selected as the synthesis route because it provides a versatile platform for designing water-soluble functional polymers. By adjusting monomer composition and introducing multifunctional units, polymer architecture and functional group distribution can be effectively regulated. BPJ was prepared through aqueous free-radical polymerization. Initially, 65.0 g of deionized water and 12.96 g of AMPS were added to a clean flask. The pH of the solution was adjusted to 7.0 with NaOH. Subsequently, 15.90 g of DMAA, 9.87 g of DMDAAC solution, and 3.67 g of AN were added. After stirring for 10 min to uniformly disperse the monomers, 0.015 g of BPEI_1800_ was introduced, followed by an additional 10 min of mixing. The reaction mixture was heated to 65 °C, and an initiator solution containing 0.15 g of V50 and 0.05 g of CAN was added. Polymerization was then conducted under nitrogen for 6 h, producing a viscous but flowable grayish-white material. The reaction duration was selected based on preliminary optimization experiments. The crude polymer was precipitated three times in ethanol and washed with deionized water to remove residual monomers and low-molecular-weight impurities. Finally, the purified product was dried at 105 °C and ground into powder to obtain BPJ. FTIR analysis verified the presence of the expected functional groups, while subsequent experiments evaluated its performance as a fluid-loss reducer.

For comparison, LPJ was synthesized using the same monomer quantities, initiator dosages, pH, reaction temperature, polymerization time, and nitrogen atmosphere as those employed for BPJ, with the sole exception that BPEI_1800_ was omitted. The resulting viscous, flowable grayish-white product was subjected to the same purification procedure, including three successive ethanol precipitations and washing with deionized water. After drying at 105 °C and pulverization, the linear polymer LPJ was obtained.

### 4.3. Characterization of BPJ

The FTIR spectra of BPJ were collected using an IRTracer-100 spectrometer (Shimadzu, Takamatsu, Japan). Before measurement, the dried polymer was thoroughly blended with anhydrous KBr and pressed into a transparent pellet. Spectral acquisition was conducted from 4000 to 400 cm^−1^ with a resolution of 0.25 cm^−1^. Thermogravimetric measurements were performed on a TGA-2 (Mettler Toledo, Zurich, Switzerland) instrument under nitrogen by increasing the temperature from 30 to 600 °C at a rate of 10 °C min^−1^. The microscopic morphology of BPJ was examined with a JEM-2100 UHR transmission electron microscope (Nippon Electron, Tokyo, Japan). Its molecular weight was measured by gel permeation chromatography using an Agilent 1260 Infinity II GPC/SEC system (Agilent Technologies, Palo Alto, CA, USA).

### 4.4. Preparation of Water-Based Drilling Fluids

Freshwater-based drilling fluids (FWBDFs): At 25 °C, 40.0 g of bentonite was dispersed in 1000 mL of deionized water. Subsequently, 1.4 g of Na_2_CO_3_ was added, and the mixture was stirred at 2000 rpm for 2 h. After standing and aging for 24 h, the freshwater-based drilling fluids (FWBDFs) were obtained.

Saltwater-based drilling fluids (SWBDFs): At 25 °C, a certain amount of NaCl and CaCl_2_ was added to the prepared freshwater-based drilling fluids, yielding the saltwater-based drilling fluids (SWBDFs).

Polymer-based drilling fluids (BPJ-FWBDFs): At 25 °C, a specific amount of BPJ was added to the FWBDFs, and the mixture was stirred at 8000 rpm for 20 min to obtain the polymer-based drilling fluids. After incorporating the LPJ, the resulting drilling fluids were denoted as LPJ-FWBDFs.

Polymer-based saltwater drilling fluids (BPJ-SWBDFs): At 25 °C, a certain amount of NaCl and CaCl_2_ was added to the polymer-based mud, then stirred at 4500 rpm for 20 min to prepare the polymer-based saltwater drilling fluids (BPJ-SWBDFs).

### 4.5. Performance Evaluation of BPJ

In accordance with API standards, the ZNN-D6 six-speed rotational viscometer (Qingdao Tongchun Petroleum Instrument Co., Ltd., Qingdao, China) was used to determine the rheological parameters of drilling fluids. Dial readings obtained at 600 and 300 rpm were designated as θ_600_ and θ_300_. Apparent viscosity (AV, mPa·s), plastic viscosity (PV, mPa·s), and yield point (YP, Pa) were calculated using Equations (1)–(3) [32]. All measurements were performed in triplicate, and the mean values are reported.

(1) $$\mathrm{AV}=0.5\;\times {\;\theta }_{600}$$

(2) $$\mathrm{PV}={\;\theta }_{600}-{\;\theta }_{300}$$

(3) $$\mathrm{YP}=0.511\;\times \;(\theta_{300}-\;\mathrm{PV})$$

API filtration loss (FL_API_) was determined at 25 °C and 0.69 ± 0.03 MPa using an SD6A medium-pressure filter press (Qingdao Tongchun Petroleum Instrument Co., Ltd., Qingdao, China). High-temperature and high-pressure filtration loss (FL_HTHP_) was measured with a GGS71-B HTHP filter press (Qingdao Tongchun Petroleum Instrument Co., Ltd., Qingdao, China). Each filtration experiment was conducted at least twice, and the mean value was reported.

For thermal-aging tests, the prepared drilling fluids were sealed in aging cells and dynamically aged in a roller oven for 16 h at the designated temperature. The cells were subsequently removed from the oven and cooled to room temperature before the rheological and filtration properties of the fluids were evaluated.

### 4.6. Mechanism Study of BPJ

Drilling fluids were centrifuged at 12,000 rpm for 20 min, and the upper clear liquid was removed. Then, the clay at the bottom was removed and vacuumed at 60 °C until its weight was constant. It was then ground into powder for testing. The adsorption capacity of BPJ on the bentonite surface was determined by the TOC-L total organic carbon analyzer (Shimadzu, Takamatsu, Japan). A 60.0 mg BPJ-containing clay sample was accurately weighed and subjected to complete combustion in an oxygen atmosphere at 900 °C. Each sample was measured five times, and the result was the average of the 5 measurements:

The drilling fluids were diluted 1000 times in deionized water. The zeta potential of drilling fluids was determined using the Nano ZS90 Zeta Potential Analyzer (Malvern Panalytical, Malvern, UK). Each sample was measured three times, and the results were averaged.

The particle size distribution was determined using a Malvern Mastersizer 3000 laser diffraction particle size analyzer (Malvern Panalytical, Malvern, UK). Each sample was analyzed in triplicate, and the average value was reported.

The API filter cake was vacuum-dried at 50 °C, and the microstructure of the cake was observed using an EVO-15/LS scanning electron microscope (Zeiss, Oberkochen, Germany).

## Figures and Tables

**Table 1:** The molecular weight of BPJ and LPJ.

Polymer	M_w_	M_n_	PDI
BPJ	463,776	257,331	1.802
LPJ	506,877	267,582	1.894

**Table 2:** Performance comparison to LPJ in FWBDFs.

Concentration (%)	Condition ^1^	AV mPa·s	PV mPa·s	YP Pa	FL_API_ mL	FL_HTHP(180 °C,3.5 MPa)_ mL
2.5% BPJ	Before aging	58.0	52.0	6.0	3.2	-
	After aging	39.0	36.0	3.0	6.0	27.8
2.5% LPJ	Before aging	106.0	96.0	10.0	3.8	-
	After aging	54.0	50.0	4.0	6.2	28.8

^1^ Aging temperature was 220 °C.

**Table 3:** Performance comparison to LPJ in SWBDFs ^2^.

Concentration (%)	Condition ^1^	AV mPa·s	PV mPa·s	YP Pa	FL_API_ mL	FL_HTHP(180 °C,3.5 MPa)_ mL
2.5% BPJ	Before aging	25.0	23.0	2.0	4.0	-
	After aging	16.0	15.0	1.0	7.0	39.8
2.5% LPJ	Before aging	46.0	43.0	3.0	4.0	-
	After aging	22.0	20.0	2.0	7.2	42.0

^1^ Aging temperature was 220 °C; ^2^ NaCl concentration in SWBDFs was 36%.

**Table 4:** Performance comparison to other fluid-loss reducers in SWBDFs ^1^.

Samples	Conditions ^2^	AV mPa·s	PV mPa·s	YP Pa	FL_API_ mL	FL_HTHP(180 °C,3.5 MPa)_ mL
2.5% BPJ	Before aging	25.0	23.0	2.0	4.0	-
	After aging	16.0	15.0	1.0	7.0	39.8
2.5% DS-1	Before aging	37.0	32.0	5.0	5.4	-
	After aging	15.0	14.0	1.0	8.8	79.8
2.5% DS-2	Before aging	53.0	47.0	6.0	8.0	-
	After aging	11.0	10.0	1.0	15.2	132.6
2.5% Driscal D	Before aging	101.0	84.0	17.0	10.4	-
	After aging	27.0	25.2	2.0	19.6	152.4
2.5% SMP-1	Before aging	46.0	38.0	8.0	21.6	-
	After aging	53.0	43.0	10.0	76.8	350.0

^1^ NaCl concentration in SWBDFs was 36%; ^2^ aging temperature was 220 °C.

## Data Availability

Data will be made available upon request. The raw experimental datasets supporting the reported results are available upon request.
